# Areas of High Biodiversity Value Evidenced by the Spatial Scaling of Phylogenetic Uniqueness

**DOI:** 10.1111/ele.70179

**Published:** 2025-07-14

**Authors:** Andrés Baselga, Ramiro Martín‐Devasa, Carola Gómez‐Rodríguez

**Affiliations:** ^1^ CRETUS, Department of Zoology, Genetics and Physical Anthropology Universidade de Santiago de Compostela Santiago de Compostela Spain; ^2^ CRETUS, Department of Functional Biology (Area of Ecology) Universidade de Santiago de Compostela Santiago de Compostela Spain

**Keywords:** distance decay models, ecological uniqueness, endemism, irreplaceability, phylogenetic dissimilarity, spatial scaling

## Abstract

Distinct biological communities have high conservation value because they harbour species that cannot be preserved elsewhere. However, community uniqueness is scale‐dependent: irreplaceability depends on whether community dissimilarity emerges at small or large spatial scales. To assess conservation value, here we integrate phylogenetic endemism with the spatial scaling of phylogenetic uniqueness in terrestrial vertebrates. We show that phylogenetic endemism is the most efficient single criterion to maximise global phylogenetic diversity within the smallest land area. Moreover, the spatial scaling of phylogenetic uniqueness allows distinguishing globally distinct but regionally less unique sites ‘(evolutionary hills)’, from highly irreplaceable sites even at small scales ‘(evolutionary islands)’, which support lower local diversity but host species that are both evolutionarily unique and threatened. This approach provides a non‐heuristic and stable baseline to identify high‐value biodiversity areas and offers a powerful tool for prioritising conservation efforts to safeguard evolutionary heritage effectively.

## Graphical abstract

Phylogenetic endemism is the most efficient single criterion to maximise global phylogenetic diversity within the smallest land area, and can be complemented with the spatial scaling of phylogenetic uniqueness which allows distinguishing globally distinct but regionally less unique sites ‘(evolutionary hills)’ from highly irreplaceable sites even at small scales ‘(evolutionary islands)’, which support lower local diversity but host species that are both evolutionarily unique and threatened.

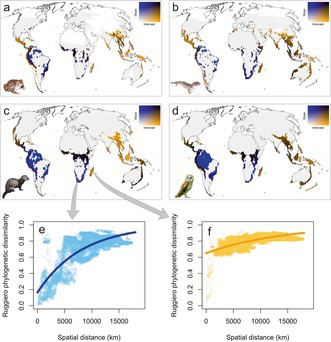

## 1 Introduction

Given the pervasive impact of human activities on biodiversity at a global scale, local community‐centred conservation goals need to be coordinated within a global strategy (Leclère et al. 2020). To this end, the Kunming‐Montreal Global Biodiversity Framework (GBF) has set the 30 × 30 target, aiming to have at least 30% of the world's land and sea areas effectively conserved and managed by 2030 (COP15 United Nations Biodiversity Conference 2022). While setting such measurable objectives is crucial for progress monitoring and evaluation, the effective allocation of conservation efforts also requires area‐based initiatives to be guided by biodiversity relevance assessments (Pimm et al. 2018; Hoffmann 2022). The need to conserve particular valuable regions, such as biodiversity hotspots (Myers et al. 2000), is well recognised and unquestioned. However, focusing solely on hyperdiverse areas may not fully maximise the protection of biological diversity relative to land area (Lamoreux et al. 2006; Pollock et al. 2017; Vimal et al. 2021). To effectively conserve global biodiversity, we also need to assess the distinctiveness of the biological communities in each region. This shifts our focus from ‘what to conserve’ to ‘where else could this set of species be conserved’, thereby accounting for biodiversity irreplaceability in a spatial context. Furthermore, the temporal dimension of irreplaceability, arising from its evolutionary history, should also weigh on biodiversity distinctiveness estimates and, in general, inform any biodiversity conservation policy (Vane‐Wright et al. 1991; Purvis, Agapow, et al. 2000; Rosauer and Mooers 2013; Swenson 2014; Cardillo 2023). Safeguarding global phylogenetic diversity (i.e., the tree of life) means preserving the legacy of a unique and non‐repeatable evolutionary process of diversification, which is crucial to ensure that future options for humanity are not compromised (Díaz et al. 2019; Owen et al. 2019; Cardillo 2023).

A distinctiveness‐focused and area‐based assessment of biodiversity value in a global context would allow conservation efforts to be informed by both the spatial and temporal irreplaceability of biodiversity. In other words, protecting global phylogenetic diversity requires a robust understanding of its spatial distribution and the complementarity among different regions. To this end, the goals of this paper are twofold. First, we aim to identify high‐value biodiversity sites through a complementarity‐based, non‐heuristic approach that is independent of regional contexts and existing conservation frameworks, such as current reserve networks. This is important because a global, stable baseline, defined purely in biological terms, would not require iterative updates and would provide an objective foundation upon which regional and local Systematic Conservation Planning can be built, in line with the two‐step approach pioneered by Vane‐Wright et al. (1991). Second, we aim to integrate evolutionary information into the identification of areas important for biodiversity conservation. While approaches like EDGE incorporate phylogenetic data, they are species‐centred (Isaac et al. 2007; Gumbs et al. 2023). Although they can also be extended to identify spatial conservation priorities (Pipins et al. 2024), EDGE‐based methods prioritise sites with many evolutionarily distinct and endangered species but, by design, do not account for the complementarity between sites, which is crucial for maximising the conservation of global phylogenetic diversity. Moreover, by explicitly modelling the scale‐dependence of phylogenetic uniqueness at a global scale, we introduce a novel biogeographic framework that distinguishes globally distinct but regionally less unique sites ‘(evolutionary hills)’ from highly irreplaceable sites that are unique even at small scales ‘(evolutionary islands)’.

Regions rich in unique biodiversity, known as areas of endemism (Orme et al. 2005; Lamoreux et al. 2006) or phylogenetic endemism (Rosauer et al. 2009; Rosauer and Jetz 2015; Daru, Farooq, et al. 2020), play a crucial role in the preservation of global biodiversity (Lamoreux et al. 2006). Endemism arises from the presence of geographically restricted species or phylogenetic clades (phylogenetic endemism), which are found nowhere else. These species are often highly vulnerable and face a greater risk of extinction (Fritz et al. 2009), making phylogenetic endemism a good proxy for both irreplaceability (i.e., the unique contribution of a site to the biodiversity of a network of areas) and vulnerability (i.e., the degree to which a site's biodiversity is threatened). Additionally, it may also serve as an approximate surrogate for community distinctiveness, although better‐suited metrics, such as ecological uniqueness or singularity, should be used to accurately measure how distinct the species community at a given site is compared to other locations. Community uniqueness typically is measured as the average dissimilarity between a focal cell and the remaining (Jurasinski et al. 2009; Mokany et al. 2022; Tsang et al. 2023), or as the Local Contribution to Beta Diversity (LCBD, Legendre and De Caceres 2013; da Silva et al. 2020; Heino et al. 2022; Luukkonen et al. 2024), which is a linear transformation of the former. When these measures incorporate evolutionary history (i.e., community phylogenetic uniqueness, Holt et al. 2013; Shooner et al. 2018; Nakamura et al. 2020), they acknowledge that communities harbouring closely related species are more similar than communities with species from distant branches of the evolutionary tree (Graham and Fine 2008).

**FIGURE 1:**
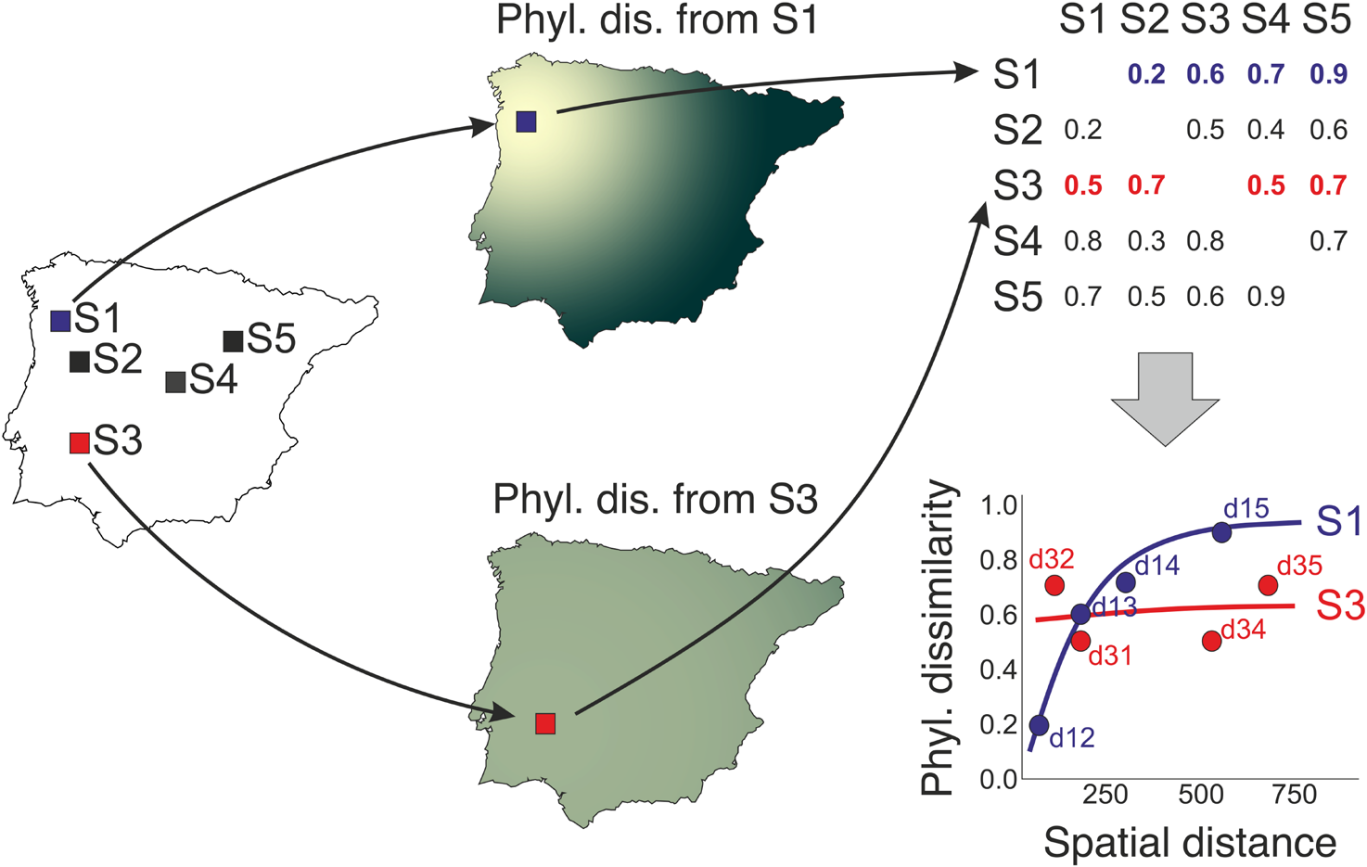
Simplified example showing how phylogenetic uniqueness is computed from the pairwise dissimilarity values between the focal sites (rows, S1‐S5) and the remaining ones (columns, S1‐S5). From left to right, the figure represents a map with five sites (S1‐S5), two maps of how phylogenetic dissimilarity increases from two example focal sites (S1 and S3) and, in the right column, a square matrix containing the dissimilarity values between pairs of biological communities of these five sites, and a plot of the relationship between these dissimilarities and spatial distance for each site (here only sites 1 and 3 are represented). Because we are interested in how unique the focal site is, dissimilarity is computed using an asymmetrical index (phylogenetic Ruggiero's dissimilarity), so the dissimilarity between S1 (focal) and S3 can be different from the similarity between S3 (focal) and S1. Therefore, focal cells must be assigned to either rows or columns (rows in this case). Therefore, the average of each row (removing the diagonal) is the phylogenetic uniqueness of each site. The relationship between pairwise dissimilarity and spatial distance accounts for the spatial scaling of phylogenetic uniqueness. In this specific example, phylogenetic uniqueness is identical (0.6) for both S1 and S3, but the spatial scaling is very different, as S1 presents a low intercept and a steep slope, while S3 presents a high intercept and a flat slope. Thus, S1 uniqueness arises from a continuous increase of dissimilarity with spatial distance (evolutionary hill), while S3 uniqueness arises from an abrupt isolation even at the closest distance (evolutionary islands).

These measures of ecological uniqueness are attributes of a focal site or cell and are therefore highly useful for conservation (Hoffmann et al. 2018), as they are straightforward to interpret and can be easily visualised on a single map (i.e., one value per site or grid cell). They are also measures of central tendency (average values) derived from complex data that may hold intrinsic value: the multiple pairwise comparisons between the focal site and all other sites. The visualisation of the more informative pairwise phylogenetic dissimilarities would result in multilayer cartographies that would require a separate map for each focal site to display the dissimilarities with all other sites (and Tsirogiannis and Sandel 2016 for phylogenetic similarity; see Gaüzère et al. 2023 for interactions similarity). In these maps, phylogenetic dissimilarity is expected to increase in a pattern radiating outward from the focal site (Figure 1). If a focal community is very similar to its nearby counterparts and markedly different to distant sites, we would observe a steep gradient, resembling a mountain peak. Conversely, a site that is highly unique even at the shortest distances would show a flatter gradient, akin to a wide plateau. To effectively capture this spatial structure of phylogenetic uniqueness while avoiding the complexity of multilayer cartographies, we propose modelling ecological uniqueness analogously to the well‐known macroecological pattern of distance‐decay of community similarity (Nekola and White 1999; Graco‐Roza et al. 2022). This approach not only allows for simple spatial representations of model parameters (e.g., slope or intercept), but also addresses a critical aspect of both phylogenetic uniqueness and phylogenetic endemism: their scale‐dependency (Daru, Farooq, et al. 2020). Values of phylogenetic endemism or average phylogenetic uniqueness can vary significantly depending on the distances considered for site comparisons (i.e., within a radius of a few hundred or thousands of kilometres). However, it is possible to overcome this limitation by explicitly modelling the increase of (phylogenetic) uniqueness with spatial distance, without being constrained by specific radii.

Here we model the global geographic patterns of phylogenetic endemism and phylogenetic uniqueness of amphibian, reptile (squamate), bird, and mammal communities. Our aim is to evaluate (i) whether criteria based on phylogenetic endemism and phylogenetic uniqueness are effective in selecting the minimum set of sites needed to protect the most evolutionary history on a global scale within the 30 × 30 target. We will also (ii) model the spatial scale dependence of site phylogenetic uniqueness, as the basis to understand whether phylogenetic uniqueness arises from continuous differentiation in space and time or from abrupt spatiotemporal discontinuities. To achieve this, we adjust inverse distance–decay models of the relationship between phylogenetic dissimilarity and spatial distance for each focal terrestrial 10,000 km^2^ cell. We focus on two parameters of these distance‐increase models of community phylogenetic uniqueness: the intercept, which indicates the degree of distinctiveness at the shortest distances, and the slope, which provides insights into the rate at which community phylogenetic uniqueness increases with spatial distance. By combining these two facets of community phylogenetic uniqueness and comparing them with phylogenetic endemism, we can identify areas of high biodiversity value and include phylogenetic uniqueness as a cornerstone of area‐based conservation goals.

## 2 Material and Methods

### 2.1 Distribution and Phylogenetic Data

Terrestrial vertebrate distributions were obtained from the IUCN Red List of Threatened Species (2022) for amphibians, reptiles and mammals, and BirdLife International (2022) for birds. We excluded from the species distributions any non‐native or extinct populations. Polygonal range maps were superimposed to an equal‐area grid with cells of 10,000 km^2^ (Sastre et al. 2009), generating four global presence‐absence tables for all the known species of amphibians, reptiles, mammals and birds. Phylogenies for the four vertebrate groups were obtained from the literature: for amphibians, reptiles, and mammals, we used the consensus trees provided by Jetz and Pyron (2018), Tonini et al. (2016) and Upham et al. (2019), respectively. For birds, we used TreeAnnotator (Bouckaert et al. 2019) to build a maximum credibility tree (median heights) from a sample of 1000 trees provided by Jetz et al. (2012). There were few taxonomic discrepancies between the IUCN distributional data and the phylogenetic data. These were solved by removing from the presence‐absence tables those species not included in the phylogenetic trees: 6 amphibians, 1 reptile, 0 mammals, and 2 birds. Finally, to avoid large effects on dissimilarity measures caused by extreme low values of species richness, all 10,000 km^2^ cells with less than five species for any given vertebrate group were removed, resulting in four datasets of 6610 species and 9320 cells (amphibians), 8720 species and 13,063 cells (reptiles), 5474 species and 15,244 cells (mammals), and 9683 species and 16,195 cells (birds).

### 2.2 Phylogenetic Uniqueness and Endemism at a Global Scale

Analyses of phylogenetic uniqueness and phylogenetic endemism were conducted separately for each vertebrate group. Phylogenetic endemism for each cell was computed following Rosauer et al. (2009) as the sum of the products of branch length and the inverse of the distribution range of all species present in a cell, using function phylo_endemism() in R package phyloregion (Daru, Karunarathne, and Schliep 2020). Phylogenetic uniqueness was computed as the average phylogenetic dissimilarity between the biotic community of a focal cell and those of the remaining cells (Figure 1).

We measured phylogenetic dissimilarity as the proportion of branch length in the phylogenetic tree that is unique to the focal cell in pairwise comparisons with the remaining ones. We refer to this dissimilarity measure as Ruggiero's phylogenetic dissimilarity (β_rlb_._phylo_), which can be computed as PD_unique_/PD_total_, with PDunique being the branch length that is unique to the focal cell when compared to another one, and PD_total_ being the total branch length of the focal cell (i.e., the sum of the unique and shared branch lengths). β_rlb_._phylo_ is an extension of the taxonomic index introduced by Ruggiero et al. (1998), and referred to as β_rlb_ in Koleff et al. (2003). Like its taxonomic version, this dissimilarity measure is not symmetrical (Koleff et al. 2003), because the proportion of unique branch length in Site 1 compared to Site 2 can be different from the proportion of unique branch length in Site 2 compared to Site 1. Thus, β_rlb.phylo_ may be different when the focal cell is either Site 1 or Site 2. This property is crucial to measure the phylogenetic uniqueness of a cell, because we are interested in the unique contribution of the focal cell, not the unique contributions of the cells to which the focal cell is compared. Computationally, the asymmetry of the index implies that pairwise dissimilarities must be stored in a square matrix in which focal cells are rows and the remaining cells are columns (Figure 1), instead of the usual triangular distance matrices. We implemented this in the new R function phylobeta.rug() (see associated code in https://doi.org/10.6084/m9.figshare.29458223.v1, and to be implemented in R package betapart (Baselga et al. 2023)). To measure the discrepancy between phylogenetic endemism and phylogenetic uniqueness, we standardised both variables in the range [0, 1] and computed the difference. This difference was then regressed against log‐transformed species richness to assess whether the discrepancy between phylogenetic endemism and phylogenetic uniqueness depends on species richness.

### 2.3 Area‐Dependent Selection Informed by Different Biodiversity Measures

We also assessed the efficiency of different biodiversity measures to maximise global phylogenetic diversity for any proportion of global area. These measures, used to prioritise conservation, included phylogenetic endemism, phylogenetic uniqueness, local species richness, local phylogenetic diversity (Faith's PD), the sum of EDGE scores, or EDGE richness. These metrics were selected because EDGE, as well as the expected loss of PD, have been adopted as complementary indicators for monitoring the progress within the Kunming–Montreal Global Biodiversity Framework (GBD), thereby integrating species' evolutionary potential and history into core biodiversity policies (Robuchon et al. 2023). In this context, EDGE‐based zones represent areas that should be prioritised as they concentrate threatened evolutionary history (Pipins et al. 2024).

To assess how global phylogenetic diversity would increase with the increase of protected area depending on the criteria used for prioritisation, we accumulated 10,000 km^2^ cells in different descending orders based on the following criteria: cell´s phylogenetic endemism, phylogenetic uniqueness, species richness, phylogenetic diversity, sum of EDGE scores and EDGE richness. We then plotted the cumulative global phylogenetic diversity (Faith's PD) encompassed by increasing proportions of global area (number of included cells/total number of cells). Faith's PD was computed using function PD() in package phyloregion (Daru, Karunarathne, and Schliep 2020). EDGE scores for all species were obtained from Gumbs et al. (2023), and lists of EDGE species were downloaded from https://www.edgeofexistence.org. From these, we computed two measures based on the EDGE approach previously used for site prioritisation: the sum of EDGE scores of all species in a given cell, and EDGE richness (i.e., the number of EDGE species present in a cell).

### 2.4 Scaling of Phylogenetic Uniqueness Based on Distance‐Increase Models

Distance‐increase models of phylogenetic uniqueness were applied to determine whether the phylogenetic uniqueness of a site arises from community differentiation processes occurring at small or large spatial scales. Two main theoretical scenarios are possible within this spatial scaling framework. First, high phylogenetic uniqueness may result from a strong, gradual increase in phylogenetic dissimilarity with distance (i.e., small intercept and steep slope), which would be indicative of long‐term but gradual, distance‐dependent eco‐evolutionary processes. Such sites would be highly distinct on a global scale but not on a regional scale, as they would be surrounded by highly similar communities. These communities or regions could be described as ‘evolutionary hills’. Conversely, high phylogenetic uniqueness may also result from communities that have been isolated over the long term, even at the shortest spatial distances, and harbour evolutionarily unique sets of species (i.e., large intercept). Such sites are highly irreplaceable because they lack alternative similar sites even nearby and can be described as ‘evolutionary islands’.

Distance‐increase models of phylogenetic uniqueness were built as follows. For each focal cell (i.e., rows in the square matrix of β_rlb_._phylo_), we fitted a negative exponential model to the relationship between phylogenetic Ruggiero's dissimilarity (*d*) and spatial distance (*s*), as *d* = 1‐ *a* * e ^b*s^, where *a* is the intercept, and *b* is the slope. The model was fitted using a GLM with log link, as implemented in a new uniqueness.model() function (see code in https://doi.org/10.6084/m9.figshare.29458223.v1, and to be implemented in R package betapart (Baselga et al. 2023)). Spatial distance between cells was computed as the geodesic distance between their centroids, using R package geodist (Padgham and Sumner 2021). These models are analogous to distance‐decay models (Nekola and White 1999; Soininen et al. 2007), but only use the dissimilarities between the focal cell and all other ones, instead of all pairwise combinations. Hence, we fit a distance‐increase model of phylogenetic uniqueness for each cell rather than a global single model for the full study area, as in distance‐decay analyses. For each focal cell, the intercept and slope of those distance‐increase models of phylogenetic uniqueness explicitly describe (i) how unique a cell is at the closest spatial distances, and (ii) the rate at which phylogenetic uniqueness increases with distance or, in other words, how phylogenetic uniqueness scales with spatial distance.

Finally, to illustrate the difference between communities characterised by high intercepts or by high slopes, we categorised the cells within the first quartile of phylogenetic endemism as evolutionary hills (i.e., those in the top quartile of phylogenetic uniqueness‐increase slope) and evolutionary islands (i.e., those in the top quartile of phylogenetic uniqueness‐increase intercepts), and assessed their differences in species richness, species average contribution to phylogenetic endemism (i.e., phylogenetic endemism/species richness), and species average EDGE score (i.e., sum of EDGE scores/species richness).

## 3 Results

### 3.1 Phylogenetic Uniqueness and Endemism at a Global Scale

**FIGURE 2:**
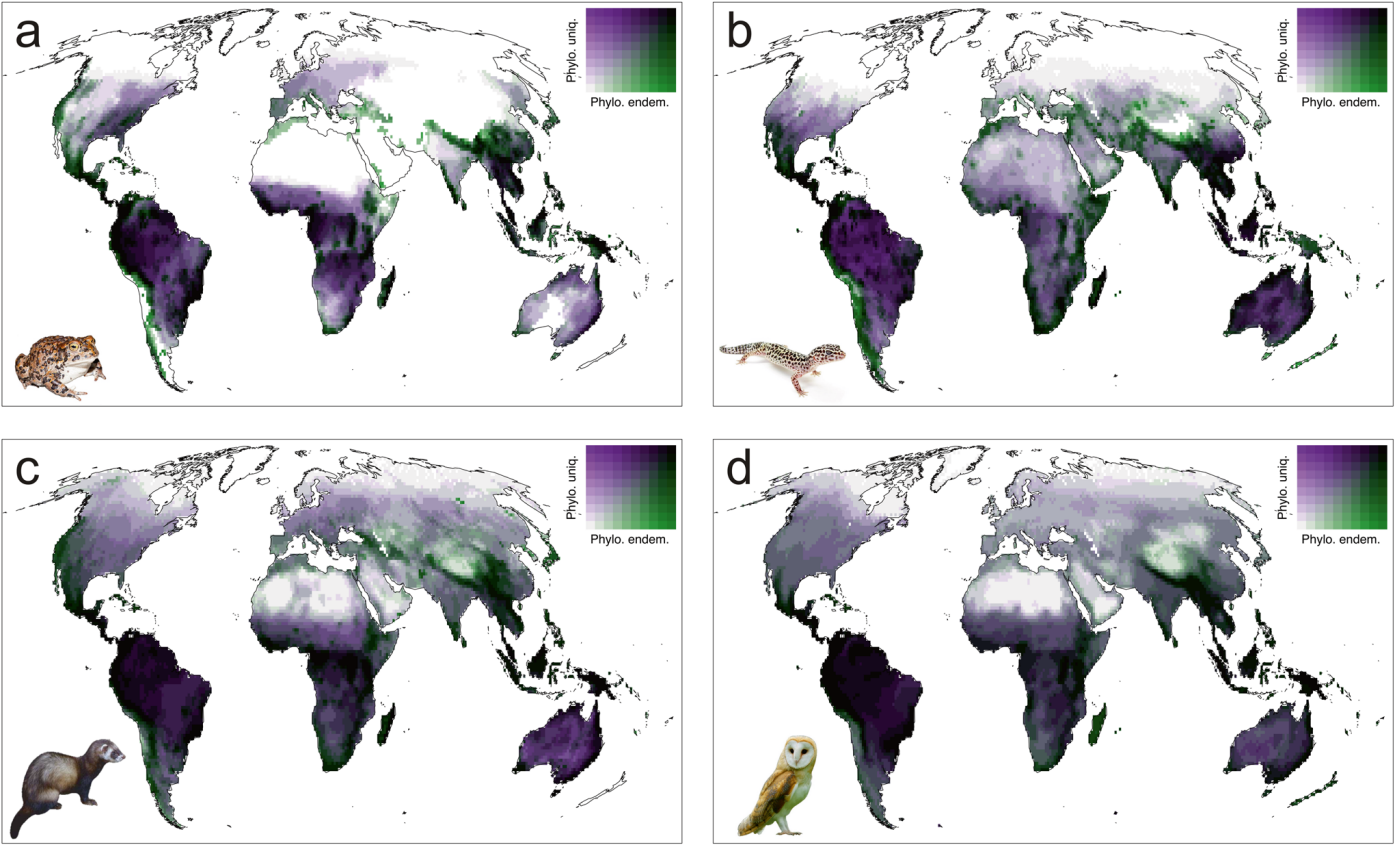
Global bivariate maps of the spatial distribution of phylogenetic uniqueness and phylogenetic endemism, for amphibians (a), reptiles (b), mammals (c) and birds (d), respectively. Green tones indicate areas of high phylogenetic endemism and low phylogenetic uniqueness, while purple tones indicate areas of high phylogenetic uniqueness and low phylogenetic endemism. Black tone indicates that both variables are high. Animal pictures were authored by Paul Maier (Yosemite toad), Matt Reinbold (leopard gecko), Malene Thyssen (European polecat) and Peter Trimming (western barn owl), all obtained from Wikimedia Commons (with CC licence).

While phylogenetic uniqueness and phylogenetic endemism are conceptually related, they are not perfect surrogates of one another (Figure 2). In general terms, their global geographic patterns were broadly similar in each studied taxa (Figures S1–S2), with both measures being highly correlated (Figure S3). Values of both measures tended to be higher in Central and South America, tropical Africa, Southeast Asia, and Australia (Figures S1–S2). However, geographic discrepancies between the two metrics evidenced that they capture different facets of the evolutionary and spatial structure of biological diversity. Figure 2 shows regions where phylogenetic endemism is higher than phylogenetic uniqueness (green tones), and vice versa (purple tones), with these discrepancies being more evident in amphibians and reptiles (Figure S3). The discrepancy between phylogenetic endemism and phylogenetic uniqueness was well explained by species richness (*r* ^2^ = 0.78 for amphibians, 0.88 for reptiles, 0.60 for mammals, and 0.34 for birds). Cross‐taxon concordance was similar for both phylogenetic uniqueness (mean Spearman correlation rho = 0.82, Table S1) and phylogenetic endemism (mean rho = 0.79, Table S2).

### 3.2 Area‐Dependent Selection Informed by Different Biodiversity Measures

**FIGURE 3:**
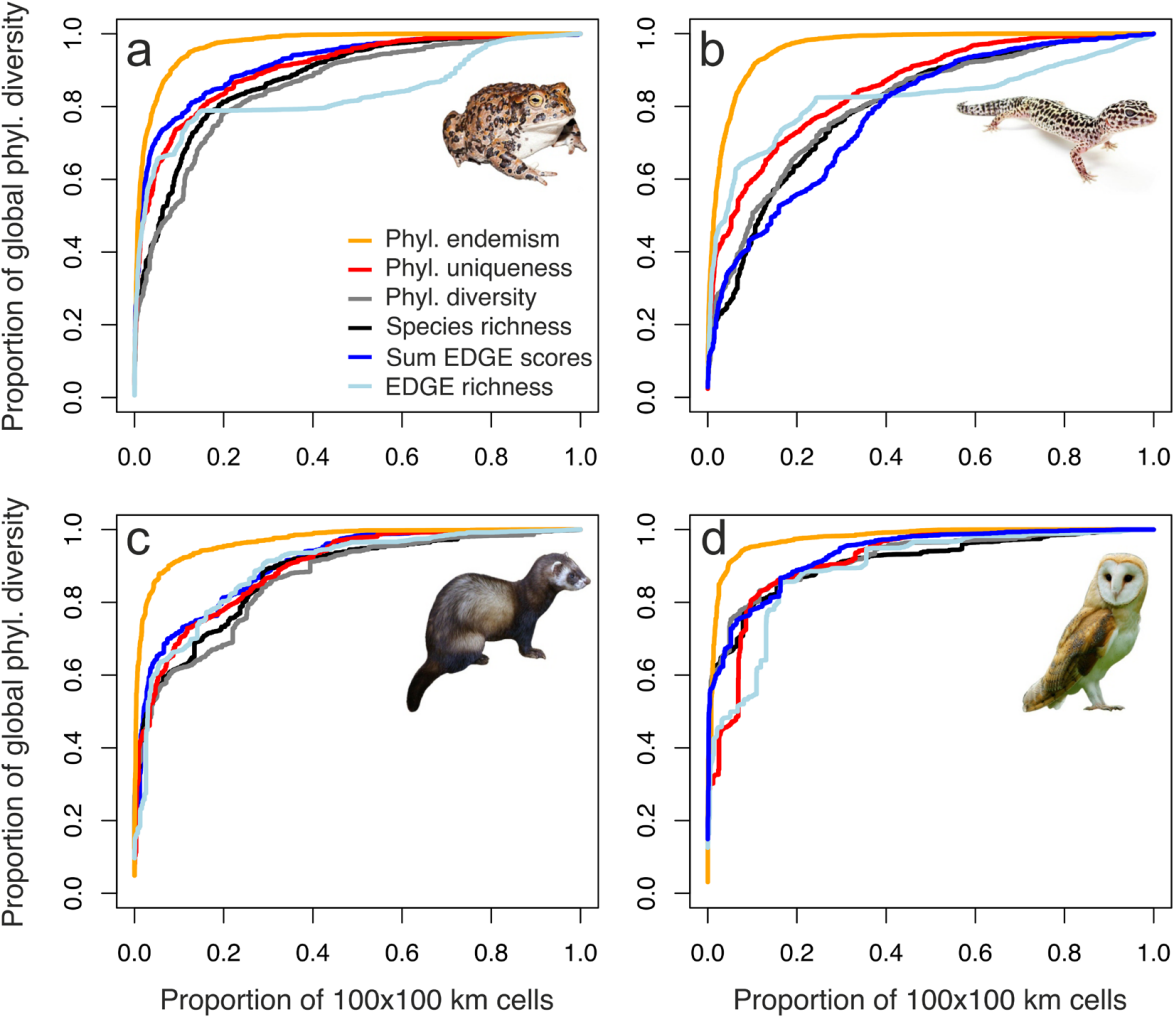
Proportion of global phylogenetic diversity (Faith's PD) covered by accumulating an increasing proportion of 10,000 km^2^ cells, prioritising them by different criteria (cell's phylogenetic endemism, phylogenetic uniqueness, phylogenetic diversity, species richness, sum of EDGE scores or EDGE richness), for amphibians (a), reptiles (b), mammals (c) and birds (d), respectively.

The studied biodiversity metrics are not equally efficient to identify areas of biodiversity importance, especially when the amount of protected area tends to be small. Accumulation curves of 10,000 km^2^ cells prioritised by their phylogenetic endemism evidenced the steepest increase in the proportion of phylogenetic diversity that would be preserved at a global scale as protected area increased (Figure 3). In contrast, prioritising cells based on average phylogenetic uniqueness offered only marginal improvements compared to species richness, phylogenetic diversity (Faith's PD) or EDGE‐based metrics. These results show that phylogenetic endemism outperforms all other single‐value criteria for site selection when the objective is maximising the total amount of global phylogenetic diversity.

**FIGURE 4:**
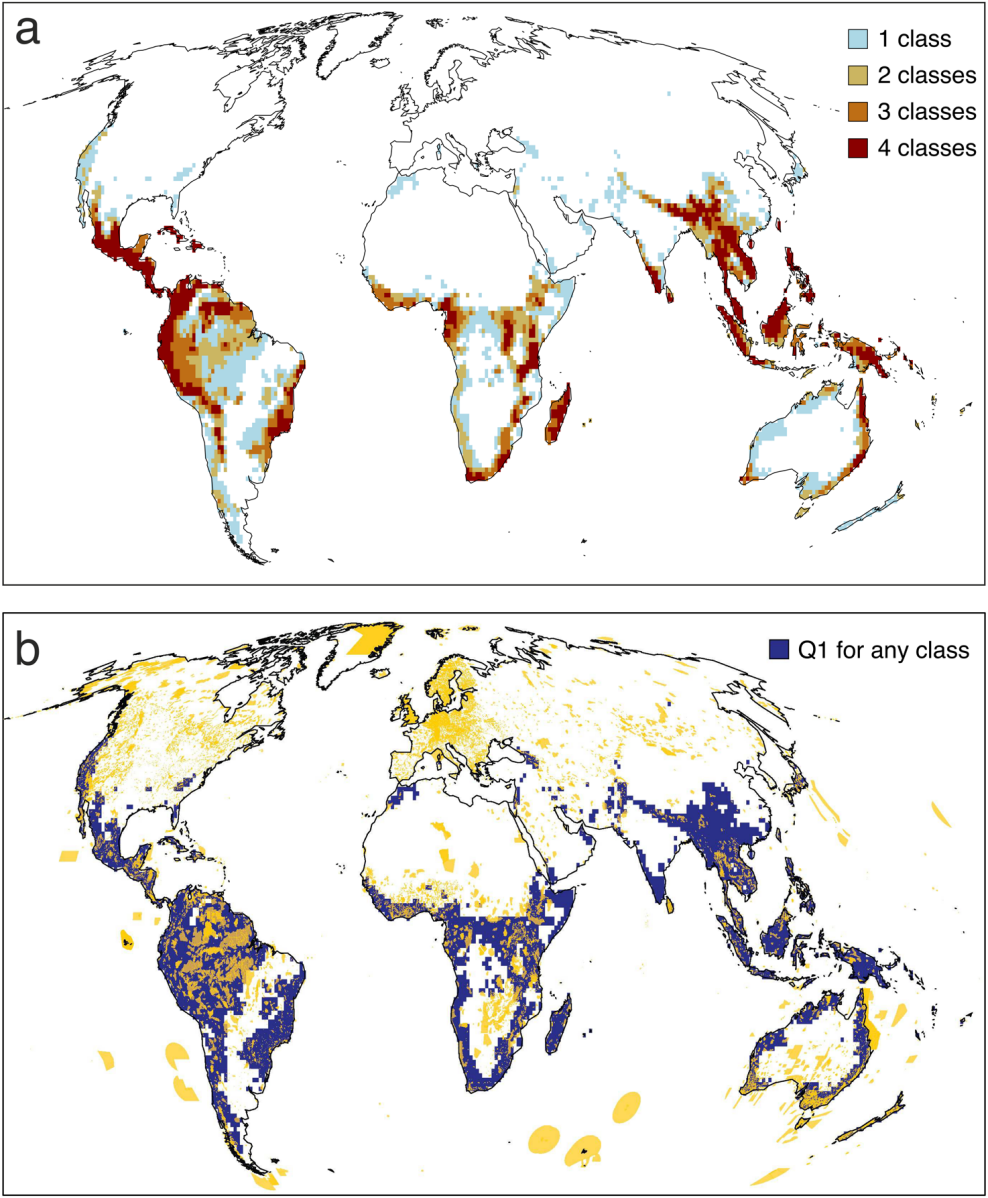
(a) Spatial distribution of 10,000 km^2^ cells that fall within the first quartile of phylogenetic endemism for four (7% of the land), three (12%), two (19%) or at least one terrestrial vertebrate class (29%). (b) Global framework of protected areas (marked in yellow) superimposed to the cells that fall within the first quartile of phylogenetic endemism for at least one of the four terrestrial vertebrate groups (marked in blue). The protected areas were extracted from the database from UNEP‐WCMC and IUCN (2023, available at: https://www.protectedplanet.net), considering UICN categories Ia, Ib and II.

Despite the broad cross‐taxon congruence, only 7% of the cells were classified within the first quartile of phylogenetic endemism for all four vertebrate classes (Figure 4). These cells, which should be considered top conservation priorities, occurred in southern Central America, western and eastern South America, western, central and south Africa, Madagascar, south India and Sri Lanka, the Himalayas, Southeast Asia and eastern Australia. Additional criteria could include considering cells that fall within the first quartile of phylogenetic endemism for at least three, two or one taxa. These would account for 12%, 19% and 29% of the area, respectively. Consequently, protecting regions in the first quartile of phylogenetic endemism for any taxa would cover approximately 30% of terrestrial land, providing an efficient solution to preserve global phylogenetic diversity within the 30 × 30 target. Notably, there is limited overlap between the current network of natural reserves and the areas identified using this first‐quartile criterion (Figure 4b).

### 3.3 Complementary Conservation Criteria Based on the Spatial Scaling of Phylogenetic Uniqueness

**FIGURE 5:**
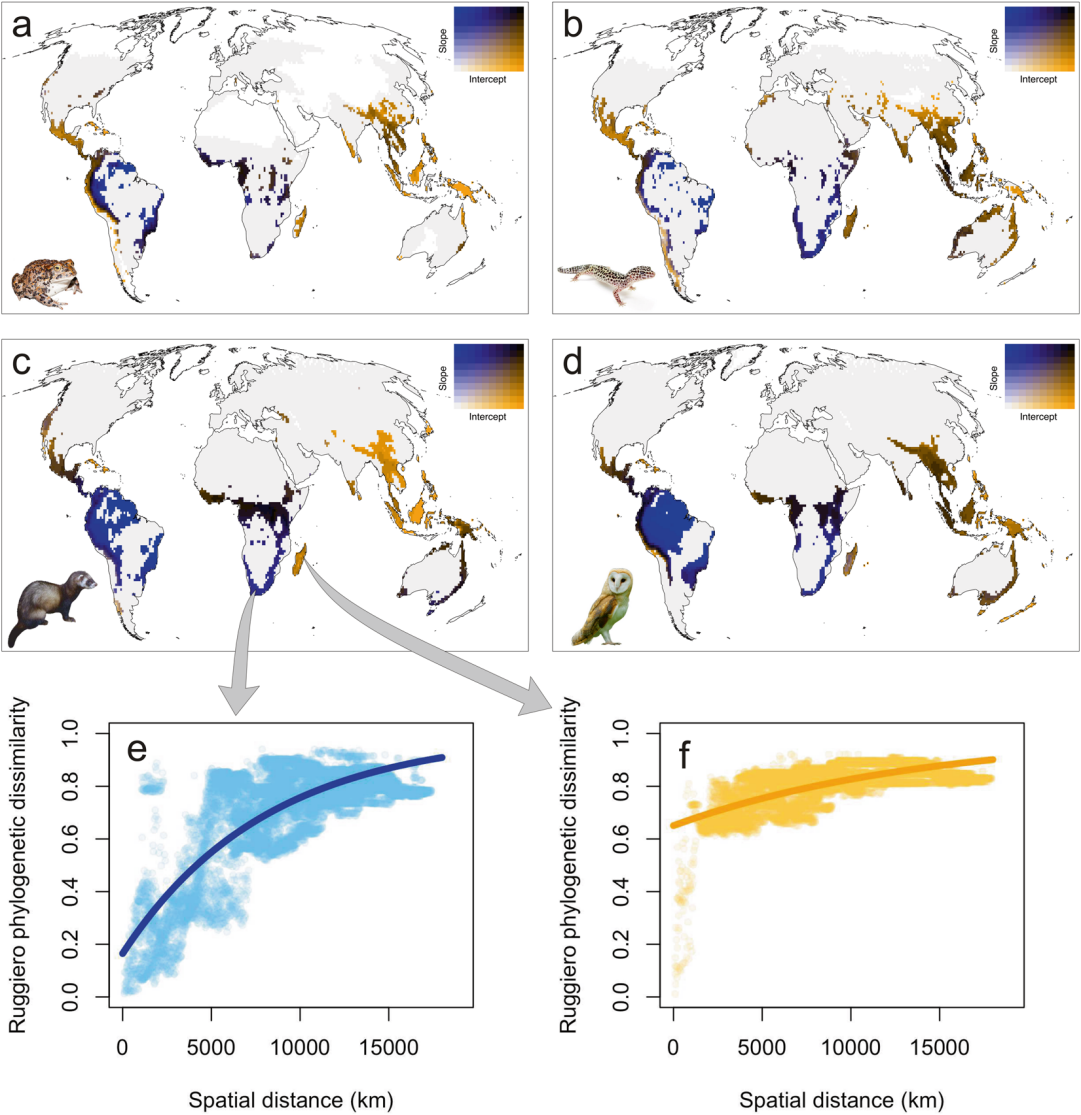
Spatial scaling of evolutionary uniqueness of 10,000 km^2^ cells within the first quartile of phylogenetic endemism, for amphibians (a), reptiles (b), mammals (c), and birds (d), respectively. Plots (e, f) show the increase of Ruggiero's phylogenetic dissimilarity with spatial distance from two focal cells, and the respective fits of uniqueness‐increase models, taken as examples of evolutionary hills (e, low intercept, high slope) and evolutionary islands (f, high intercept, flat slope).

When we applied our spatial scaling framework, distance‐increase models of phylogenetic uniqueness showed that high average phylogenetic uniqueness may result from both ‘evolutionary hills’ and ‘evolutionary islands’, as the spatial patterns of intercepts and slopes were largely decoupled. Steep slopes were observed in Africa and South America, except the Andes, while high intercepts were observed in southern North America, Central America, the Mediterranean, Southeast Asia and Australia (Figure S4). Interestingly, cells within the first quartile of phylogenetic endemism also exhibited widely different patterns in the spatial scaling of phylogenetic uniqueness, characterised by steep slopes, high intercepts or both (Figure 5). However, only high intercepts indicate communities that are highly unique even within their closer region and are therefore more irreplaceable and of higher biodiversity relevance from a conservation perspective. These evolutionary islands, which fall within the first quartile of phylogenetic endemism, are broadly geographically concordant across the four vertebrate groups: Mexico and Central America, the Andes, Equatorial and South Africa, Madagascar, southern India, South East Asia (both continental and insular) and eastern Australia.

**FIGURE 6:**
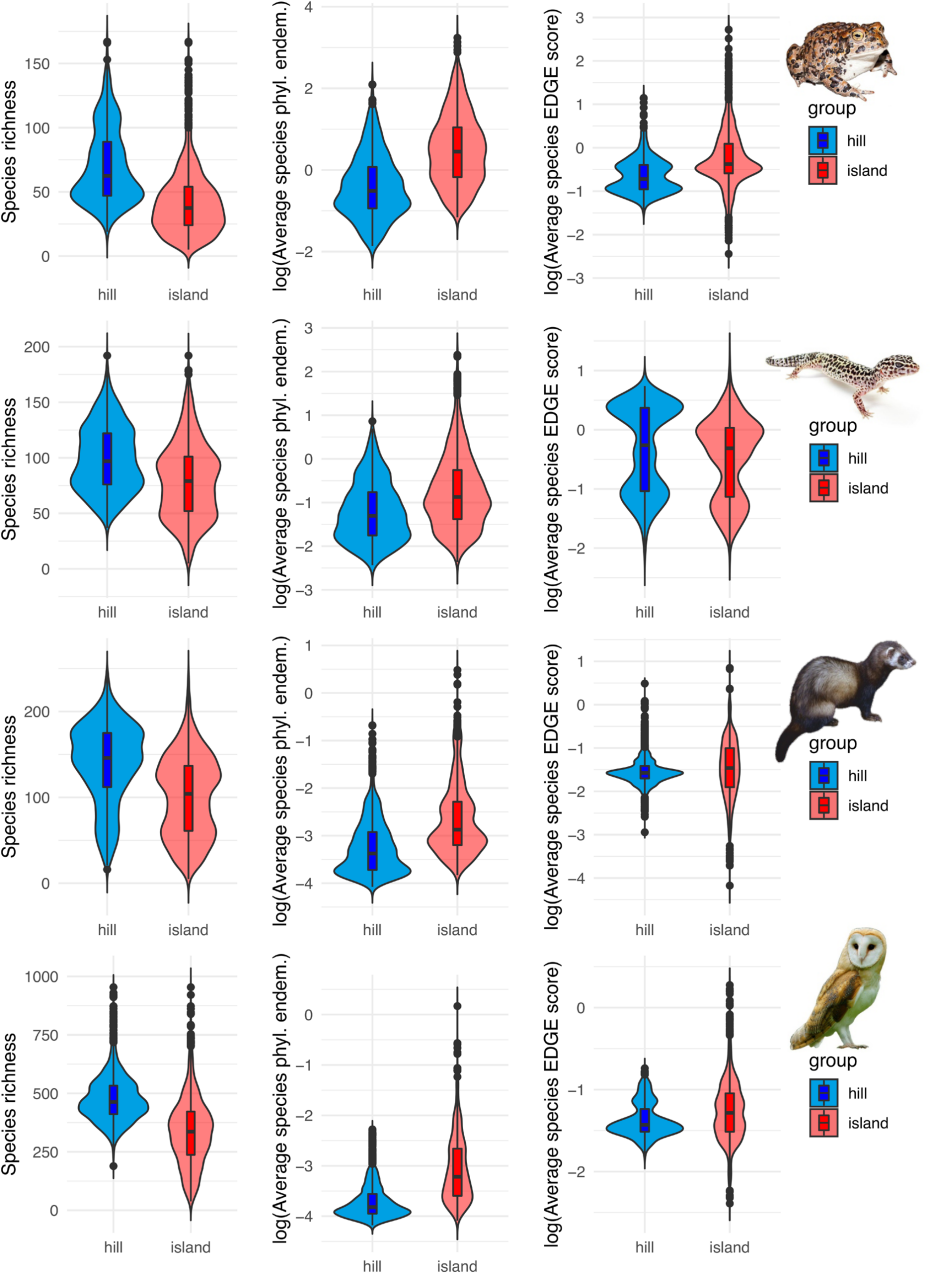
Contrasting distributions of species richness (left column), average species contribution to phylogenetic endemism (central column), and average species EDGE score (right column) between evolutionary hills (blue) and evolutionary islands (red) for amphibians, reptiles, mammals and birds (first, second, third and fourth row, respectively). The evolutionary hills considered here are the cells falling in the first quartile of phylogenetic endemism and the first quartile of slopes in the uniqueness‐increase models. The evolutionary islands are the cells falling in the first quartile of phylogenetic endemism and the first quartile of intercepts in the uniqueness‐increase models.

When we compared evolutionary hills versus evolutionary islands within the first quartile of phylogenetic endemism (Figure 6), it turned out that evolutionary hills tended to have higher species richness, while the species average contribution to phylogenetic endemism and EDGE scores were higher in evolutionary islands. All differences were significant when assessed with Wilcoxon tests (all *p* < 0.00001).

## 4 Discussion

Regions with unique sets of species are of paramount conservation importance at a global scale. Here we complement standard metrics, such as phylogenetic endemism or phylogenetic uniqueness, with models accounting for the spatial scaling of phylogenetic uniqueness for each site in the globe (i.e., distance‐increase models of phylogenetic uniqueness). This approach allows us to differentiate between sites that are highly distinct on a global scale but not on a regional scale (i.e., ‘evolutionary hills’) and highly irreplaceable sites, even at the smallest scale (i.e., ‘evolutionary islands’). Distance‐increase models of phylogenetic uniqueness simultaneously account for both the evolutionary history and spatial distribution of multiple species, providing integrative metrics of biodiversity relevance across spatial and temporal scales. This is crucial because conservation actions must necessarily originate locally (Wyborn and Evans 2021), yet a site's conservation value is often tied to the irreplaceability of its biological communities (Pressey et al. 1994; Brooks et al. 2006). Studies like this one, based on large grid cells, should therefore be seen as a preliminary step in identifying regions that may merit further fine‐scale conservation assessments (i.e., the initial round of the two‐step process proposed by Vane‐Wright et al. 1991). This is particularly relevant because the phylogenetic diversity being lost at a global scale constitutes the most irreplaceable component of biological diversity: it derives from the evolutionary process of branching of the Tree of Life, which is both unique and non‐repeatable. Unlike taxonomic and functional diversity, which may rebound after mass extinctions (Edie et al. 2018; Song et al. 2018), lost phylogenetic lineages are gone permanently. Thus, the limited reserve coverage of areas of high biodiversity value is cause for serious concern. Nonetheless, we also stress that the regions that we identify as having high biodiversity value are by no means the only ones deserving conservation efforts.

We provide a scale‐free assessment of biodiversity relevance by integrating phylogenetic endemism with the spatial structure of phylogenetic uniqueness. The identification of areas of high biodiversity value is usually based on local or regional diversity measures such as species richness (Myers et al. 2000), endemism (Lamoreux et al. 2006), phylogenetic diversity (Tietje et al. 2023), phylogenetic endemism (Murali et al. 2021; Cai et al. 2023), species of special interest (Ward et al. 2020), or threatened phylogenetic history (i.e., the sum of EDGE scores, Pipins et al. 2024). In contrast, our proposal offers a macroecological perspective based on dissimilarity metrics and hence rooted in beta diversity, which inherently aligns with the key conservation principle of complementarity (Justus and Sarkar 2002). However, it should not be confused with other complementarity‐based tools like systematic conservation planning algorithms, which heuristically select priority areas to maximise biodiversity coverage at the minimal cost. Rather than serving as an optimisation framework, our hotspot delimitation captures spatial variation in phylogenetic uniqueness and endemism, which could be used to identify broad regions that should be further assessed to determine which areas within those regions should be prioritised, for example following a complementary‐based iterative algorithm (Shipley and McGuire 2022). As such, it provides a valuable foundation for identifying (phylogenetic) Key Biodiversity Areas (PD‐KBAs), while presenting phylogenetic information in a format that is both accessible and informative for conservation policy implementations (Cardillo 2023).

Our scale‐free assessment of biodiversity relevance has two main advantages. First, unlike EDGE scores, phylogenetic endemism and phylogenetic uniqueness do not require any assessment of conservation status, so they could be readily applied to other biological groups for which this information is not available. Second, by integrating the classical alpha–beta–gamma diversity partitioning framework into conservation strategies (Socolar et al. 2016), we provide a unified framework that explicitly addresses the spatial structure and scaling of biological diversity, aligning conservation efforts with the goal of maximising the global preservation of biodiversity. For example, areas with high values of threatened phylogenetic history (sum of EDGE scores) largely correspond to areas of high species richness (Pipins et al. 2024) in tropical South America, Africa and Asia (Figure S5). However, this criterion misses several areas that we identify as key for preserving global phylogenetic diversity, both due to their high phylogenetic endemism and the fact that they are identified as evolutionary islands (i.e., large areas in Central America, the Andes or New Guinea, for example). Due to these discrepancies, we suggest that phylogenetic endemism should be used as a primary criterion for maximising global phylogenetic diversity within the smallest possible area. To complement this, phylogenetic uniqueness offers two additional advantages: (i) it can be used to identify areas harbouring communities that, while not necessarily highly diverse, are nonetheless evolutionarily distinctive, and (ii) it allows the assessment of how phylogenetic uniqueness scales with spatial scale.

Distance‐increase models of phylogenetic uniqueness enable the detection of emergent properties of biodiversity patterns that arise only when examined at a macroecological scale. Decoupling phylogenetic uniqueness at smaller scales (i.e., high intercept, probably due to long‐term isolation) from phylogenetic uniqueness arising due to gradual phylogenetic turnover across space (i.e., high slope) provides important and novel insight into the phylogenetic and biogeographic processes that contribute to the irreplaceability of specific sites. Focusing on areas of high phylogenetic endemism, important differences between two types of communities (evolutionary hills vs. evolutionary islands) are revealed. Evolutionary hills tend to have higher species richness, while phylogenetic islands harbour communities that are less diverse but composed of more unique species. There is broad cross‐taxon congruence in the distribution of these evolutionary hills (e.g., the Amazon and southern Africa) and islands (e.g., central America, Madagascar or southeast Asia), suggesting that shared biogeographic and historical processes have driven contrasting levels of isolation between these regions. Evolutionary islands are regions that have experienced long‐term isolation, thus harbouring clades that are not found even in nearby regions. Some of these regions are geographic islands (e.g., the Caribbean, Madagascar, Southeast Asia), while others are continental areas that have remained isolated due to paleogeographic history (e.g., Central America, Kirby and MacFadden 2005), or the presence of habitat islands linked to topographic features, as in Mexico (Marshall and Liebherr 2000), the Andes (Särkinen et al. 2012), the Western Ghats (Bose et al. 2016) or the Himalayas (Wambulwa et al. 2021). Indeed, the association between evolutionary islands and mountain systems, particularly in tropical and subtropical regions, is in alignment with previous findings that link endemism with elevation, a relationship that tends to increase towards the equator (Steinbauer et al. 2016). In contrast, evolutionary hills are found in widespread continental areas with tropical and subtropical climates, such as South America and southern Africa.

Finally, it is also worth noting that phylogenetic uniqueness (and phylogenetic endemism) serves as an indirect indicator of vulnerability, as spatial range size is often linked to extinction risk (Purvis, Gittleman, et al. 2000; Davidson et al. 2009). Importantly, while phylogenetic diversity measures are usually correlated with species richness and highly congruent among taxonomic groups, our findings reveal that the spatial scaling of phylogenetic uniqueness varies among taxa, thus providing unique information not previously available. Using the spatial scaling of phylogenetic uniqueness to assess the biodiversity value of local communities aligns with the criteria established in the Key Biodiversity Areas framework (Eken et al. 2004) and its extension of Phylogenetic Key Biodiversity Areas (Faith 2015): irreplaceability and vulnerability. This approach offers a valuable perspective for the implementation of the 30 × 30 target of the Kunming‐Montreal Global Biodiversity Framework (GBF), as it would allow preserving highly valuable sites with minimum protected area.

## Author Contributions

A.B. and C.G.‐R. designed the study; R.M.‐D. processed the data; A.B. performed the analyses; C.G.‐R. led the writing of the manuscript, with input from A.B.

## Supporting information


**Data S1.** Supporting Information.

## Data Availability

All data and code supporting the results have been archived at https://doi.org/10.6084/m9.figshare.29458223.v1.
