# Supplementary material for: Areas of High Biodiversity Value Evidenced by the Spatial Scaling of Phylogenetic Uniqueness
Source: Ecol Lett. 2025 Jul 14;28(7):e70179. doi: 10.1111/ele.70179 (PMC12258097; doi:10.1111/ele.70179)
Supplement: Supplementary file 1 — Data S1. Supporting Information. [file ELE-28-0-s001.docx]

Supplementary material for:

**Areas of high biodiversity value evidenced by the spatial scaling of phylogenetic uniqueness**

Andrés Baselga, Ramiro Martín-Devasa, Carola Gómez-Rodríguez

Table S1. Spearman rank correlation between average phylogenetic uniqueness of the four terrestrial vertebrate groups. Correlation was computed using all complete pairs of observations on each pair of vertebrate groups.

|  | Amphibians | Reptiles | Mammals | Birds |
| --- | --- | --- | --- | --- |
| Amphibians |  | 0.68 | 0.81 | 0.81 |
| Reptiles | 0.68 |  | 0.83 | 0.84 |
| Mammals | 0.81 | 0.83 |  | 0.95 |
| Birds | 0.81 | 0.84 | 0.95 |  |

**Table S2.** Spearman rank correlation between phylogenetic endemism of the four terrestrial vertebrate groups. Correlation was computed using all complete pairs of observations on each pair of vertebrate groups.

|  | Amphibians | Reptiles | Mammals | Birds |
| --- | --- | --- | --- | --- |
| Amphibians |  | 0.64 | 0.82 | 0.80 |
| Reptiles | 0.64 |  | 0.78 | 0.79 |
| Mammals | 0.82 | 0.78 |  | 0.93 |
| Birds | 0.80 | 0.79 | 0.93 |  |

**Figure S1.** Global map of average phylogenetic uniqueness, for amphibians (a), reptiles (b), mammals (c), and birds (d), respectively.

**Figure S2.** Global map of phylogenetic endemism, for amphibians (a), reptiles (b), mammals (c), and birds (d), respectively.

**Figure S3.** Relationship between average phylogenetic uniqueness (y-axis) and the logarithm of phylogenetic endemism (x-axis) for amphibians, reptiles, mammals and birds. Each dot is a 10000 km^2^ cell and the density of dots is represented by the colour gradient, from blue (low density) to red (high density). The Spearman rank correlation (rho) between both variables is shown for each group.

**Figure S4.** Bivariate maps of the spatial distribution of slopes and intercepts of the uniqueness-increase models, which characterise the spatial scaling of evolutionary uniqueness in each 10000 km^2^ cell, for amphibians (a), reptiles (b), mammals (c), and birds (d), respectively. Blue tones identify evolutionary hills, i.e. sites that are unique in a gradual manner, while orange tones identify evolutionary islands, i.e. sites that are unique even at shorter distances.

Fig. S5. Global map of phylogenetic the sum of EDGE scores, for amphibians (a), reptiles (b), mammals (c), and birds (d), respectively.
